# T-DM1 versus pertuzumab, trastuzumab and a taxane as first-line therapy of early-relapsed HER2-positive metastatic breast cancer: an Italian multicenter observational study

**DOI:** 10.1016/j.esmoop.2021.100099

**Published:** 2021-04-02

**Authors:** F. Schettini, B. Conte, G. Buono, P. De Placido, S. Parola, G. Griguolo, A. Fabi, C. Bighin, F. Riccardi, D. Cianniello, M. De Laurentiis, F. Puglisi, G. Pelizzari, M. Bonotto, S. Russo, A. Frassoldati, A. Pazzola, F. Montemurro, M. Lambertini, V. Guarneri, F. Cognetti, M. Locci, D. Generali, P. Conte, S. De Placido, M. Giuliano, G. Arpino, L. Del Mastro

**Affiliations:** 1 Department of Clinical Medicine and Surgery, University of Naples ‘Federico II’, Naples, Italy; 2 Translational Genomics and Targeted Therapies in Solid Tumors Group, August Pi i Sunyer Biomedical Research Institute (IDIBAPS), Barcelona, Spain; 3 SOLTI Breast Cancer Research Group, Barcelona, Spain; 4 Medical Oncology Unit 2, IRCCS Ospedale Policlinico San Martino, Genoa, Italy; 5 Oncology Unit, San Rocco Hospital, Sessa Aurunca, Italy; 6 Division of Oncology 2, Istituto Oncologico Veneto IRCCS, Padua, Italy; 7 Department of Surgery, Oncology and Gastroenterology, University of Padova, Padua, Italy; 8 Division of Medical Oncology 1, IRCCS Regina Elena National Cancer Institute, Rome, Italy; 9 Medical Oncology, ‘A. Cardarelli’ Hospital, Naples, Italy; 10 National Cancer Institute Fondazione ‘G. Pascale’, Naples, Italy; 11 Department of Medicine (DAME), University of Udine, Udine, Italy; 12 Department of Clinical Oncology, CRO Aviano National Cancer Institute, Aviano, Italy; 13 Department of Oncology, ASUFC University Hospital, Udine, Italy; 14 Oncology Unit, University Hospital St. Anna, Ferrara, Italy; 15 Division of Medical Oncology, AOU Sassari, Sassari, Italy; 16 Depertment of Medical Oncology, Candiolo Cancer Institute, Candiolo, Italy; 17 Department of Internal Medicine and Medical Specialties (DiMI), School of Medicine, University of Genova, Genoa, Italy; 18 Department of Medical Oncology, U.O.C. Clinica di Oncologia Medica, IRCCS Ospedale Policlinico San Martino, Genoa, Italy; 19 Department of Clinic and Molecular Medicine, ‘La Sapienza’ University of Rome, Rome, Italy; 20 Department of Neuroscience, Reproductive Medicine, Odontostomatology, University of Naples ‘Federico II’, Naples, Italy; 21 Breast Cancer Unit, Azienda Socio Sanitaria Territoriale di Cremona, Cremona, Italy; 22 Department of Medical, Surgery and Health Sciences, University of Trieste, Trieste, Italy; 23 U.O.S.D. Breast Unit, IRCCS Ospedale Policlinico San Martino, Genoa, Italy

**Keywords:** T-DM1, pertuzumab, trastuzumab, breast cancer, HER2, first-line

## Abstract

**Background:**

The current standard first-line treatment of human epidermal growth factor receptor 2 (HER2)-positive (+) metastatic breast cancer is the combination of pertuzumab, trastuzumab and a taxane (P + T + taxane), while standard second-line is ado-trastuzumab-emtansine (T-DM1). The registration trial of pertuzumab, however, did not include early-relapsing patients, defined as patients experiencing tumor relapse ≤12 months from the end of (neo)adjuvant anti-HER2 therapy. Conversely, the pivotal trial of T-DM1 included some patients relapsing ≤6 months after the end of (neo)adjuvant trastuzumab. Thus, a proportion of early-relapsing patients are currently eligible to receive T-DM1 as first-line treatment. Nevertheless, no direct comparison exists between the two regimens in this clinical setting.

**Patients and methods:**

We retrospectively compared T-DM1 versus P + T + taxane as first-line treatment in two cohorts of early-relapsing patients in an Italian ‘real-world’ setting, involving 14 public health care institutions. The primary endpoint was progression-free survival. Secondary endpoints included patients' characterization, overall survival and post-progression survival. Univariate and multivariate analyses were carried out. All tests were two-sided and a *P* ≤ 0.05 was considered statistically significant.

**Results:**

Among 1252 screened patients, 75 met the inclusion criteria. Forty-four (58.7%) received P + T + taxane and 31 (41.3%) received T-DM1. The two cohorts showed similar characteristics of aggressiveness and no significant differences in treatment history. T-DM1, compared with P + T + taxane was associated with worse progression-free survival (adjusted hazard ratio: 2.26, 95% confidence interval: 1.13-4.52, *P* = 0.021) and overall survival (adjusted hazard ratio: 3.95, 95% confidence interval: 1.38-11.32, *P* = 0.010), irrespective of previous (neo)adjuvant treatment, age, hormone receptors status, time-to-relapse (≤6 months or within 6-12 months) and presence of visceral/brain metastases. No differences were observed in post-progression survival (*P* = 0.095).

**Conclusions:**

Our study suggests superiority for P + T + taxane over T-DM1 as up-front treatment of early-relapsing HER2+ metastatic breast cancer, which merits further assessment in larger and prospective trials.

## Highlights

• This is the first study comparing pertuzumab + trastuzumab + taxane (P + T + taxane) with T-DM1 in early-relapsing HER2+ MBC.
• The majority of early-relapsing HER2+ MBC have high-grade, node-positive, large primary tumors.
• First-line T-DM1 compared with P + T + taxane is associated with worse progression-free survival.
• First-line T-DM1 compared with P + T + taxane is associated with worse overall survival.
• Post-progression survival does not differ between the two treatments cohorts.

## Introduction

Human epidermal growth factor receptor 2 (HER2)-positive (+) breast cancer, irrespective of hormone receptor status, accounts for 11%-30% of all breast tumors.^1^ Survival for metastatic disease has risen from a median of ∼20 months before the introduction of anti-HER2 targeted agents, to ∼45 months.^2^^,^^3^ Current first- and second-line therapeutic standards are, respectively, the combination of pertuzumab + trastuzumab (P + T) + docetaxel/paclitaxel and the antibody–drug conjugate ado-trastuzumab-emtansine (T-DM1).^4^, ^5^, ^6^ Pertuzumab-containing regimens were approved following significant progression-free survival (PFS) and overall survival (OS) improvements over trastuzumab-based regimens, as observed in the CLEOPATRA phase III pivotal trial and further ‘real-world’ studies.^5^, ^6^, ^7^, ^8^, ^9^, ^10^, ^11^ T-DM1 was approved for the second and further lines after showing significant PFS and OS improvements over lapatinib + capecitabine and treatment of physician's choice in the EMILIA and TH3RESA phase III trials, respectively.^4^^,^^12^, ^13^, ^14^

Early-stage HER2+ tumors usually respond very well to anti-HER2-based (neo)adjuvant treatments, with estimated mean annual hazard of recurrence of only ∼3%-4% in years 0-5 after systemic therapy.^15^ Unfortunately, a small proportion do not respond properly and relapse within 12 months from the end or during (neo)adjuvant anti-HER2-based therapy.^16^, ^17^, ^18^ These early-relapsing cases have been associated with worse prognosis^17^^,^^19^ and were not represented in the CLEOPATRA trial.^5^ Nevertheless pertuzumab-based regimens were approved as the first-line option independently of the time-to-relapse (TTR). Conversely, a small proportion (15.6%) of patients with early-relapsing tumors [though only within 6 months from the end or during (neo)adjuvant trastuzumab treatment] were included in the EMILIA trial.^4^ For this reason, T-DM1 was also approved by international regulatory agencies in this subset as first-line treatment, with some guidelines also extending the recommendation to early relapses between 6 and 12 months from the end of previous therapy.^20^ Thus, an undefined number of patients with early-relapsing HER2+ metastatic breast cancer receives T-DM1 in first-line.

At present, there is no published study reporting a direct comparison between P + T + taxane and T-DM1 in the first-line setting of early-relapsing HER2+ metastatic breast cancer patients and limited evidence exists about the optimal treatment strategy for this understudied subpopulation. Our aim was to compare the two available first-line treatment options in a one-to-one fashion and provide potentially useful evidence for the management of this neglected subgroup of patients.

## Methods

### Study design and patient population

This was an observational retrospective multicenter study involving 14 different Italian health care facilities (Academic Hospitals, Research Institutes and other Public Hospitals).

Patients' data were retrieved from databases previously described in other published observational studies.^9^^,^^21^, ^22^, ^23^ For the purpose of this study, only patients who had received T-DM1 or P + T and a taxane in the first-line setting of metastatic HER2+ breast cancer, outside of an interventional clinical trial and irrespective from gender, menopausal status and hormone receptor status, were included. All patients had to be affected by early-relapsing disease, defined as a distant or locally inoperable relapse which occurred during (neo)adjuvant systemic therapy with trastuzumab or chemotherapy (CT), or within 12 months from the last administration of trastuzumab. Also, patients who experienced a disease progression in <12 months after a post-neoadjuvant treatment surgery not followed by other systemic treatments were included. Study patients had been diagnosed with disease relapse between 2013 and 2019. No patient received pertuzumab and T-DM1 in an early setting, these treatments being unavailable at the time.

All patients provided informed consent for participating in the respective observational studies focused on their anticancer treatment strategies. All studies were approved by the institutional review boards of each participating center.^9^^,^^21^, ^22^, ^23^

### Study objectives

The primary objective of this study was to compare the efficacy of T-DM1 versus P + T + taxane in the first-line treatment of early-relapsing metastatic HER2+ breast cancer, in terms of PFS.

Secondary objectives were:

• To compare patient and tumor characteristics between the two treatment cohorts;
• To compare the efficacy of the two regimens in terms of OS;
• To compare post-progression survival (PPS) of the two patient cohorts after first-line;

PFS was defined as the time (in months) elapsing from the start of first-line treatment to the date of the first disease progression or death from any cause, whichever occurred first. OS was defined as the time between the start of first-line treatment and the date of death from any cause. PPS was defined as the time elapsing from when the first tumor progression occurred during the first-line treatment, to the date of patients' death from any cause.

### Data extraction

Three researchers (FS, BC and GG) extracted data regarding patient and tumor characteristics, TTR, distant recurrence patterns and treatment history for metastatic and early-stage disease. The TTR was defined as the time from the end of the last (neo)adjuvant systemic therapy (including trastuzumab or CT, but not hormone therapy) or the date of surgical intervention (in case the tumor relapsed after surgery) to the time of distant or locally inoperable recurrence.

Hormone receptor status and HER2 expression were determined by local pathologists in each participating center, according to American Society of Clinical Oncology/College of American Pathologists (ASCO/CAP) guidelines.^24^, ^25^, ^26^ Tumor restaging was assessed locally by treating physicians, according to their clinical practice. Nevertheless, both pertuzumab and T-DM1 are prescribed in Italy through a central electronic registry of the Agenzia Italiana del Farmaco, which mandates tumor restaging every three cycles/9 weeks, otherwise not permitting further drug prescriptions. Therefore, all patients in this study had similar restaging timing.

### Statistical analyses

Patient and tumor characteristics and responses were compared with Fisher's exact test, χ^2^ test and Wilcoxon rank sum test with continuity correction, where appropriate. Survival curves were estimated by the Kaplan–Meier method and differences between curves were evaluated by the log-rank test. Patients alive were censored at the date of the last follow-up. Cox regression models were applied to estimate univariate hazard ratios (HR) with their 95% confidence intervals (CI) and conduct multivariate analyses for PFS and OS. Apart from first-line treatment, age at first-line (continuous), hormone receptor status (positive versus negative), (neo)adjuvant CT and (neo)adjuvant trastuzumab administration (yes versus no), TTR (6-12 months versus ≤6), and the presence/absence of visceral and brain metastases at relapse were adopted as Cox models' covariates.

The proportional hazards assumption for univariate and multivariate Cox regression models was tested using correlation coefficients between transformed survival times and scaled Schoenfeld residuals^27^ and further checked with the smoothed plots of Schoenfeld residuals.^28^ All tests were two-sided and *P* ≤ 0.05 was considered statistically significant.

All analyses were carried out with R version 3.6.1 for Mac OS X and Microsoft Excel® for Mac OS X version 16.42.

## Results

### Population characteristics

For this retrospective, multicenter observational study, we included patients from several previously described Italian databases of metastatic breast cancer.^9^^,^^21^, ^22^, ^23^ We screened 1252 patients with HER2+ metastatic breast cancer treated at 14 Italian Healthcare Institutions between 2013 (year of pertuzumab and T-DM1 approval in Italy) and 2019. Among them, only 75 (6.0%) met the inclusion criteria in terms of treatment choice, TTR and availability of sufficient data for analyses (Supplementary Figure S1, available at https://doi.org/10.1016/j.esmoop.2021.100099). Forty-four (58.7%) patients received P + T + taxane and 31 (41.3%) T-DM1 as first-line therapy. The majority of baseline tumor and patient characteristics, as well as the proportion of baseline visceral (liver/lung) and central nervous system (CNS) metastases did not differ between the two cohorts, except for a higher proportion of tumors in the T-DM1 cohort with Ki67 ≥ 20% (96.3% versus 77.5%, *P* = 0.034) and G3 (96.0% versus 77.8%, *P* = 0.048). All demographics are fully reported in Table 1. All patients were women and the majority of them were postmenopausal (65.8%). Most tumors were ductal (95.3%) and hormone receptor-positive (53.3%). Additionally, both cohorts showed a majority of baseline tumors with aggressive features, namely G3 (85.2%), positive axillary lymph-nodes (N1-3: 72.6%) and large primary tumor size (T2-4: 71.0%). With respect to previous treatments, there were no significant differences in the proportion of patients receiving or not (neo)adjuvant CT, hormone therapy and trastuzumab (*P* = 0.816, *P* = 0.431, *P* = 0.197, respectively). Compared with P + T + taxane, however, the T-DM1 cohort received more anthracycline + taxane-based regimens in the early setting (78.6% versus 55.3%, *P* = 0.006) and presented a significantly higher proportion of patients with a TTR ≤6 months (90.3% versus 56.8%, *P* = 0.002). Treatment history is reported in detail in Table 2.

**Table 1:** Patient and basal tumor demographics

Patient and baseline tumor characteristics	First-line treatment group				*P*
	P + T + taxane		T-DM1		
	*N*	%	*N*	%	
	44	58.7	31	41.3	
Age at first-line					
Median (years)	54	—	51	—	0.256
IQR (years)	47-59	—	41-58	—	
Total	44	100.0	31	100.0	
Sex					
Female	44	100.0	31	100.0	—
Male	0	0.0	0	0.0	
Total	44	100.0	31	100.0	
Menopausal status					
Pre/perimenopausal	15	34.1	10	34.5	0.972
Postmenopausal	29	65.9	19	65.5	
Total	44	100.0	29	93.5	
Histotype					
Ductal	41	97.6	20	90.9	0.228
Lobular	1	2.4	2	9.1	
Other	0	0.0	0	0.0	
Total	42	95.5	22	71.0	
T					
1	12	32.4	6	24.0	0.557
2	15	40.5	10	40.0	
3	3	8.1	5	20.0	
4	7	18.9	4	16.0	
Total	37	84.1	25	80.6	
N					
0	11	29.7	6	24.0	0.966
1	14	37.8	10	40.0	
2	7	18.9	3	12.0	
3	5	13.5	6	24.0	
Total	37	84.1	25	80.6	
Hormone receptor status					
Positive	21	47.7	19	61.3	0.246
Negative	23	52.3	12	38.7	
Total	44	100.0	31	100.0	
Grading					
G1-2	8	22.2	1	4.0	**0.048**
G3	28	77.8	24	96.0	
Total	36	81.8	25	80.6	
Ki67					
<20%	9	22.5	1	3.7	**0.034**
≥20%	31	77.5	26	96.3	
Total	40	90.9	27	87.1	
Visceral metastases *ab initio*					
Visceral (liver/lung)	18	42.9	16	53.3	0.380
Non-visceral (other from liver/lung)	24	57.1	14	46.7	
Total	42	95.5	30	96.8	
CNS metastases *ab initio*					
CNS	12	27.3	6	20.0	0.474
Non-CNS	32	72.7	24	80.0	
Total	44	100.0	30	96.8	

CNS, central nervous system; IQR, interquartile range; P + T, pertuzumab + trastuzumab.

Bold values indicate significant *P* values.

**Table 2:** Treatment history

Treatment history	First-line treatment group				*P*
	P + T + taxane		T-DM1		
	*N*	%	*N*	%	
	44	58.7	31	41.3	
(Neo)adjuvant HT					
Yes	17	38.6	13	48.1	0.431
No	27	61.4	14	51.9	
Total	44	100.0	27	87.1	
HT type					
Tamoxifen ± GnRHa	8	47.1	5	45.5	0.934
AI ± GnRHa	9	52.9	6	54.5	
Other	0	0.0	0	0.0	
Total	17	100.0	11	84.6	
(Neo)adjuvant CT					
Yes	39	88.6	28	90.3	0.816
No	5	11.4	3	9.7	
Total	44	100.0	31	100.0	
CT type					
Anthracyclines without taxanes	4	10.5	0	0.0	**0.006**
Taxanes without anthracyclines	4	10.5	6	21.4	
Anthracyclines + taxanes	21	55.3	22	78.6	
Other	9	23.7	0	0.0	
Total	38	97.4	28	100.0	
(Neo)adjuvant trastuzumab					
Yes	33	75.0	27	87.1	0.197
No	11	25.0	4	12.9	
Total	44	100.0	31	100.0	
TTR					
≤6 months	25	56.8	28	90.3	**0.002**
>6 months and ≤12 months	19	43.2	3	9.7	
Total	44	100.0	31	100.0	
Therapy after first-line PD					
T-DM1	14	58.3	0	0.0	—
Lapatinib + capecitabine	2	8.3	15	65.2	
Other	2	8.3	4	17.4	
Unknown at last FU	4	16.7	0	0.0	
None (death during first-line)	2	8.3	4	17.4	

AI, aromatase inhibitor; CT, chemotherapy; FU, follow-up; GnRHa, gonadotropin-releasing hormone analogue; HT, hormone therapy; P + T, pertuzumab + trastuzumab; PD, progression of disease; TTR, time-to-relapse.

Bold values indicate significant *P* values.

It is worth noting that no significant differences were observed in the majority of clinicopathological features and treatment history, when comparing patients who experienced a very early relapse (TTR ≤6 months) with the ones with TTR of 6-12 months. The former were only significantly younger (*P* = 0.025) and received more T-DM1 (*P* = 0.002) as first-line treatment, consequently showing different proportions of second-line treatments (*P* = 0.007). All features are reported in Supplementary Table S1, available at https://doi.org/10.1016/j.esmoop.2021.100099.

### Univariate analyses of survival

**Figure 1:**
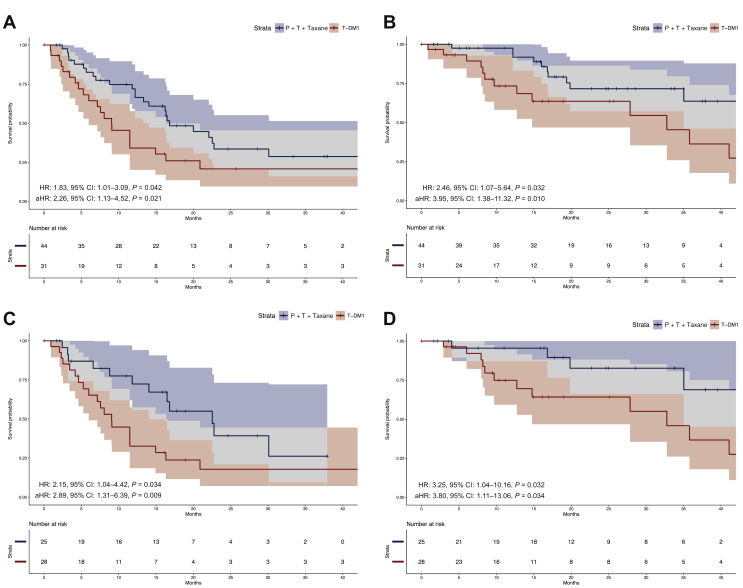
Kaplan–Meier curves of progression-free survival and overall survival. Kaplan–Meier curves with respective 95% CIs of progression-free survival (A and C) and overall survival (B and D) in the overall population and in the subpopulation with time-to-relapse ≤6 months, respectively. aHR, adjusted hazard ratio; CI, confidence interval; HR, hazard ratio; P + T, pertuzumab + trastuzumab.

The median follow-up of the overall population was 24.9 months (95% CI: 17.8-33.8 months). A shorter PFS was observed for the T-DM1 cohort, compared with the P + T {median PFS: 9.1 months (95% CI: 6.0-16.32 months) versus 16.8 months [95% CI: 14.0 to not available (NA)], HR: 1.83, 95% CI: 1.01-3.29, *P* = 0.042; Figure 1A}. Similarly, first-line T-DM1 was associated with a significantly shorter OS compared with P + T + taxane [median OS: 32.8 months (95% CI: 14.8 months to NA) versus not reached (95% CI: 35.0 months to NA), HR: 2.46, 95% CI: 1.07-5.64, *P* = 0.032; Figure 1B]. Conversely, PPS did not differ significantly between the two cohorts (*P* = 0.095; Supplementary Figure S2, available at https://doi.org/10.1016/j.esmoop.2021.100099).

Due to a numeric imbalance between the two cohorts with respect to the number of patients with TTR between 6 and 12 months (19 patients in the P + T + taxane cohort versus 3 in the T-DM1 cohort), we carried out a comparison restricted to the TTR ≤6 months population (25 patients in the P + T + taxane cohort versus 28 in the T-DM1 cohort). T-DM1 was associated with a statistically significant worse outcome in comparison with P + T + taxane in both PFS (HR: 2.15, 95% CI: 1.04-4.42, *P* = 0.034) and OS (HR: 3.25, 95% CI: 1.04-10.16, *P* = 0.032) also in this case (Figure 1C-D). We also conducted an analysis restricted to the P + T + taxane cohort, to compare the performance of the pertuzumab-containing regimen in patients with TTR between 6 and 12 months versus TTR ≤6 months. No significant differences in both PFS (*P* = 0.477) and OS (*P* = 0.211) were observed (Supplementary Figure S3, available at https://doi.org/10.1016/j.esmoop.2021.100099).

### Multivariate analyses of survival

**Figure 2:**
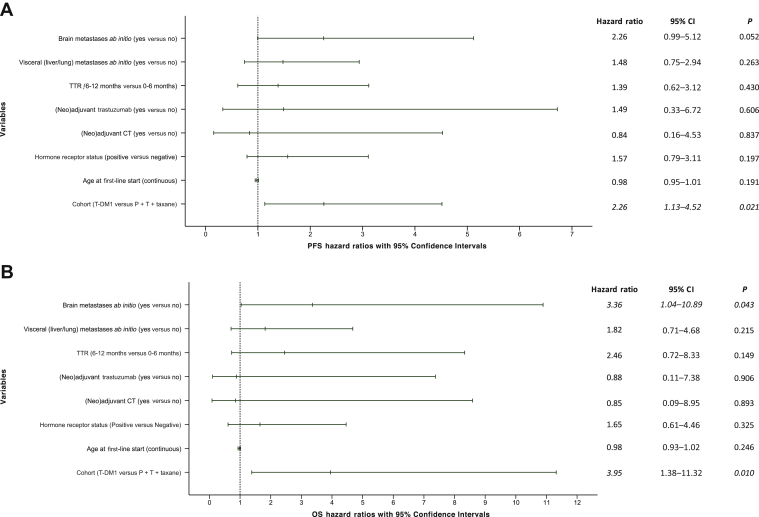
Forest plot of multivariate analysis of PFS and OS for the overall population. On the left side the variables (groups of comparisons) of the multivariate model for (A) PFS and (B) OS are listed. On the right side, the adjusted hazard ratios with 95% confidence intervals and respective *P* values are reported. CI, confidence interval; CT, chemotherapy; OS, overall survival; P + T, pertuzumab + trastuzumab; PFS, progression-free survival; TTR, time-to-relapse.

In a multivariate model including age at first-line, hormone receptor status (positive versus negative), (neo)adjuvant CT and trastuzumab administration (yes versus no), TTR (6-12 months versus ≤6), and the presence/absence of visceral and brain metastases at relapse, T-DM1 was associated with significantly worse PFS, when compared with P + T + taxane (adjusted HR: 2.26, 95% CI: 1.13-4.52, *P* = 0.021). Similarly for OS (adjusted HR: 3.95, 95% CI: 1.38-11.32, *P* = 0.010). The presence of brain metastases at baseline was associated with a marginally non-significant worse PFS (*P* = 0.052) and a significantly worse OS, independently from treatment and other covariates (*P* = 0.043). None of the other models' covariates displayed significant results in PFS and OS (Figure 2). When restricting the analysis to the population with TTR ≤6 months, T-DM1 was associated with a statistically significant worse PFS (adjusted HR: 2.89, 95 % CI: 1.31-6.39, *P* = 0.009) and OS (adjusted HR: 3.80, 95% CI: 1.11-13.06, *P* = 0.034), irrespective of the same covariates. In this case, patients with visceral and brain metastases were associated with worse PFS (*P* = 0.038 and *P* = 0.021), but not OS (*P* = 0.062 and *P* = 0.242, respectively), independently from all other covariates (Table 3).

**Table 3:** Multivariable analyses for the subpopulation with TTR ≤6 months

Variables	HR	Inferior 95% CI	Superior 95% CI	*P*
Progression-free survival				
Cohort (T-DM1 versus P + T + taxane)	2.89	1.31	6.39	**0.009**
Age at first-line start (continuous)	0.99	0.95	1.04	0.778
Hormone receptor status (positive versus negative)	1.85	0.76	4.52	0.175
(Neo)adjuvant CT (yes versus no)	1.17	0.18	7.45	0.867
(Neo)adjuvant trastuzumab (yes versus no)	1.25	0.26	5.86	0.782
Visceral (liver/lung) metastases *ab initio* (yes versus no)	2.40	1.05	5.46	**0.038**
Brain metastases *ab initio* (yes versus no)	3.24	1.20	8.76	**0.021**
Overall survival				
Cohort (T-DM1 versus P + T + taxane)	3.80	1.11	13.06	**0.034**
Age at first-line start (continuous)	0.98	0.92	1.04	0.506
Hormone receptor status (positive versus negative)	1.95	0.54	7.03	0.306
(Neo)adjuvant CT (yes versus no)	1.22	0.10	15.22	0.876
(Neo)adjuvant trastuzumab (yes versus no)	0.60	0.06	5.62	0.652
Visceral (liver/lung) metastases *ab initio* (yes versus no)	2.93	0.95	9.04	0.062
Brain metastases *ab initio* (yes versus no)	2.47	0.54	11.22	0.242

CI, confidence interval; CT, chemotherapy; HR, hazard ratio; P + T, pertuzumab + trastuzumab; TTR, time-to-relapse.

Bold values indicate significant *P* values.

### Validation of the proportional hazards assumption

The proportional hazards assumption for univariate PFS and OS analyses according to treatment cohort was not violated (*P* = 0.110 and *P* = 0.550, respectively). The assumption was also confirmed for both PFS and OS multivariate models (global *P* = 0.067 and *P* = 0.210, respectively). All the covariates included in both models passed the Schoenfeld tests (Supplementary Figures S4 and S5, and Supplementary Table S1, available at https://doi.org/10.1016/j.esmoop.2021.100099). Finally, the proportional hazards assumption was confirmed for PPS according to treatment cohort (*P* = 0.250) as well (Supplementary Figure S5, available at https://doi.org/10.1016/j.esmoop.2021.100099).

## Discussion

We retrospectively assessed the efficacy of T-DM1 compared with P + T + taxane in a cohort of metastatic HER2+ breast cancer patients, relapsed within 12 months from the end of (neo)adjuvant systemic therapy with CT ± trastuzumab. T-DM1 was associated with significantly inferior PFS and OS compared with pertuzumab-based regimens, irrespective of age, hormone receptor status, (neo)adjuvant treatment, TTR and the presence of visceral and brain metastases when starting first-line therapy. Importantly, PPS did not differ between the two cohorts, further suggesting the importance of the first-line therapy previously administered.

Early-relapsing HER2+ tumors account for a relatively low proportion of patients treated with surgery and (neo)adjuvant systemic therapies.^17^^,^^18^ In fact, since the introduction of trastuzumab as adjuvant treatment, the incidence of tumor relapses and mortality rates significantly decreased. Indeed, only 3%-18% of trastuzumab-treated patients relapse during the following 10 years, nowadays.^29^, ^30^, ^31^ Among these patients, only a small subgroup relapses very quickly, and the most appropriate therapeutic strategy for this scenario is still unknown. A recent *post hoc* analysis from the Adjuvant Lapatinib and/or Trastuzumab Treatment Optimisation (ALTTO) trial described the clinicopathological differences of HER2+ breast cancer patients relapsing within and after 12 months from the end of the adjuvant anti-HER2 therapy and showed that a treatment-free interval of <12 months has a strong negative prognostic impact, suggesting implications for the subsequent management of the metastatic setting.^19^ Patients with a TTR ≤12 months were younger, showed bigger primary lesions, more axillary node-positive primaries and more high-grade (G3) tumors compared with the population with a TTR > 12 months.^19^ Notably, our population showed very similar findings, with primary tumors characterized by a high proportion of high-grade, axillary node-positive and large primary tumor size in early stage, with no significant differences between tumors with TTR ≤6 months or between 6 and 12 months. Additionally, almost half of our patients presented at metastatic diagnosis visceral (liver/lung) involvement (∼47%) and around a quarter (∼24%) showed CNS metastases. Similarly, the ALTTO analysis in patients with a TTR ≤12 months showed brain metastases among the first sites of relapse in ∼25% of cases.^19^

As observed for other patient subgroups,^32^ early-relapsing HER2+ metastatic tumors are frequently underrepresented in clinical trials. At present, the only study of current standard metastatic anti-HER2 regimens containing a proportion of patients with very early relapse is the EMILIA.^4^ For this reason, T-DM1 was also approved for the first-line treatment of early-relapsing HER2+ advanced disease.^33^^,^^34^ Additionally, the latest ASCO guidelines recommend T-DM1 also for patients relapsing in the time interval of 6-12 months from previous (neo)adjuvant therapy, regrouping all early-relapsing patients in one single category (i.e. ≤12 months).^20^ The EMILIA trial, however, only included a small proportion of patients relapsing during or within 6 months from the end of adjuvant trastuzumab, did not enroll patients with TTR of 6-12 months and did not provide data specific to the early-relapsing subpopulation included (i.e. exact number, characteristics, response to therapy). It is worth noting that the most solid evidence regarding T-DM1 efficacy in the first-line comes from the MARIANNE phase III randomized, controlled trial (RCT), which compared T-DM1, alone or combined with pertuzumab, with trastuzumab + taxane. This study, despite showing the non-inferiority of the experimental arms to the former therapeutic standard, failed to show the superiority of first-line T-DM1 ± pertuzumab to trastuzumab + taxane. Importantly, the reference arm is no more the therapeutic standard in this setting, since pertuzumab was not included.^35^ In fact, the standard first-line option for HER2+ metastatic breast cancer is usually represented by P + T + taxane, following the PFS and OS results for the CLEOPATRA trial.^5^^,^^7^ Unfortunately, patients with relapse ≤12 months were not included in this study at all.^5^ Nevertheless, no restriction based on TTR has been produced by regulatory agencies for pertuzumab first-line use.

Notably, albeit two small observational reports and a first/second-line single-arm phase II trial showed some efficacy for pertuzumab in advanced lines,^36^, ^37^, ^38^ no RCT has demonstrated a significant efficacy of pertuzumab-based regimens after first-line, so far. Indeed, a second-line phase III RCT evaluating the addition of pertuzumab to trastuzumab and capecitabine did not improve PFS, when compared with trastuzumab + capecitabine alone.^39^ For this reason, in metastatic disease, pertuzumab is only approved in the first-line setting. On the contrary, T-DM1 clearly demonstrated its superiority over the therapeutic standard in terms of both PFS and OS in second and further lines in two phase III RCTs^13^^,^^14^ and several ‘real-world’ observational studies, also in patients pretreated with P + T + taxanes.^21^^,^^23^^,^^40^, ^41^, ^42^ Therefore, apart from prescribing caveats, the currently available evidence is clear in highlighting a major efficacy of the pertuzumab-based regimens in first-line and of T-DM1 in second and further lines. In this perspective, our study shed a light on the specific subset of early-relapsing patients and suggests a potential superiority of P + T + taxane over T-DM1 as first-line treatment of this prognostically unfavored subgroup.

The main limitations of our study rely in its retrospective nature and the limited sample size, although the rarity of the early-relapsing population makes it difficult to recruit a large amount of patients. Furthermore, only half of the original databases reported information on both HER2 immunohistochemical score and *in situ* hybridization status, though the latter was only reported as amplified/not amplified. This is major, considering that inter-pathologists agreement on HER2 scoring has been demonstrated to be potentially suboptimal, especially for 1+ and 2+ immunohistochemical score.^43^ Additionally, interpretation of *in situ* hybridization results has changed through time^25^^,^^26^; however, the possibility to homogeneously retrospectively reassess all samples for HER2 status according to the latest ASCO/CAP guidelines was not feasible.^25^ Another issue is that patients pertained to different databases and thus were not necessarily consecutively enrolled. Moreover, patients did not receive neoadjuvant pertuzumab or post-neoadjuvant T-DM1, since these treatments were not available before 2019. In fact, the therapeutic scenario of early-stage HER2+ breast tumors is in constant evolution, with pertuzumab having been approved for the neoadjuvant setting in combination with trastuzumab, and also in the adjuvant setting, for selected high-risk patients.^44^, ^45^, ^46^, ^47^ Similarly, adjuvant T-DM1 has been approved for patients treated with neoadjuvant anti-HER2-based regimens that do not achieve a pathologic complete response.^30^^,^^33^^,^^34^ Whether these novel early-setting regimens can negatively affect subsequent metastatic treatments for relapsing tumors is yet to be clarified. Recently, emerging novel anti-HER2 therapeutics (e.g. trastuzumab deruxtecan, margetuximab, tucatinib) have proven to be effective in highly pretreated patients, including those treated with pertuzumab and T-DM1 in an advanced setting and are FDA approved.^48^, ^49^, ^50^ It is possible that these novel drugs might gain a major role in earlier lines, especially in potential anti-HER2-resistant early-relapsing tumors. This is an issue that will need to be addressed in future studies.

Notably, the T-DM1 cohort was characterized by a higher number of patients relapsing within 6 months from previous early-stage treatments, compared with the P + T cohort (Table 2). This imbalance might have influenced the unfavorable outcome of the T-DM1 cohort. However, at the same time, it is important to highlight that some of the main known features usually affecting survival did not differ (i.e. age, previous treatment history, the proportion of node-positive tumors and big primary lesions). Furthermore, multivariate models were used to limit, as much as possible, potential imbalances related to the lack of randomization. Moreover, when we restricted the analysis to the subset of patients with TTR ≤6 months, the association of T-DM1 with worse PFS and OS was retained. Importantly, the result was strengthened by the similar PPS observed between the two treatment cohorts, since this suggests that the OS prolongation observed in the pertuzumab-treated group was likely to be ascribable to the first-line treatment effect. Importantly, the median PFS of P + T + taxane and T-DM1 were very similar to what was observed in their respective pivotal trials (16.8 and 9.1 months versus 18.7 and 9.6 months, respectively).^4^^,^^5^ This is reassuring with respect to the plausibility and coherence of our retrospective results with the available literature evidence. Furthermore, no differences were observed when comparing the patients with TTR of 6-12 months versus ≤6 months within the pertuzumab-treated cohort, supporting a similar treatment effect in all early-relapsing patients. Finally, the viability of the Cox regression models was also assessed through Schoenfeld residuals significance tests and graphic visual inspection, which did not raise any specific concern.

In conclusion, P + T + taxane appears to be associated with a better long-term outcome compared with T-DM1 in HER2+ metastatic breast tumors relapsed during or within 12 months from the administration of (neo)adjuvant CT and/or trastuzumab. Although larger and prospective studies are warranted to draw definitive conclusions, these results, taken together with the available evidence regarding pertuzumab and T-DM1 in first and further lines, as well as the prescription limitations concerning pertuzumab, may support the use of P + T + taxane as up-front treatment of metastatic HER2+ breast tumors independently from TTR.

## References

[1] Schettini F., Pascual T., Conte B. (2020). HER2-enriched subtype and pathological complete response in HER2-positive breast cancer: a systematic review and meta-analysis. Cancer Treat Rev.

[2] Slamon D.J., Leyland-Jones B., Shak S. (2001). Use of chemotherapy plus a monoclonal antibody against HER2 for metastatic breast cancer that overexpresses HER2. N Engl J Med.

[3] Gobbini E., Ezzalfani M., Dieras V. (2018). Time trends of overall survival among metastatic breast cancer patients in the real-life ESME cohort. Eur J Cancer.

[4] Verma S., Miles D., Gianni L. (2012). Trastuzumab emtansine for HER2-positive advanced breast cancer. N Engl J Med.

[5] Baselga J., Cortés J., Kim S.-B. (2012). Pertuzumab plus trastuzumab plus docetaxel for metastatic breast cancer. N Engl J Med.

[6] Bachelot T., Ciruelos E., Schneeweiss A. (2019). Preliminary safety and efficacy of first-line pertuzumab combined with trastuzumab and taxane therapy for HER2-positive locally recurrent or metastatic breast cancer (PERUSE). Ann Oncol.

[7] Swain S.M., Miles D., Kim S.-B. (2020). Pertuzumab, trastuzumab, and docetaxel for HER2-positive metastatic breast cancer (CLEOPATRA): end-of-study results from a double-blind, randomised, placebo-controlled, phase 3 study. Lancet Oncol.

[8] Swain S.M., Kim S.-B., Cortés J. (2013). Pertuzumab, trastuzumab, and docetaxel for HER2-positive metastatic breast cancer (CLEOPATRA study): overall survival results from a randomised, double-blind, placebo-controlled, phase 3 study. Lancet Oncol.

[9] De Placido S., Giuliano M., Schettini F. (2018). Human epidermal growth factor receptor 2 dual blockade with trastuzumab and pertuzumab in real life: Italian clinical practice versus the CLEOPATRA trial results. Breast.

[10] Polito L., Shim J., Du Toit Y., Do T., Knott A., Thibaut Sanglier T. (2020). Abstract P1-18-14: Use of pertuzumab in combination with taxanes for HER2-positive metastatic breast cancer (MBC): analysis of US electronic health records. Cancer Res.

[11] Gamucci T., Mentuccia L., Sperduti I. (2017). Efficacy of pertuzumab in combination with trastuzumab and a taxane in first-line treatment for metastatic breast cancer (MBC): a multicenter, retrospective, observational study. J Clin Oncol.

[12] Diéras V., Miles D., Verma S. (2017). Trastuzumab emtansine versus capecitabine plus lapatinib in patients with previously treated HER2-positive advanced breast cancer (EMILIA): a descriptive analysis of final overall survival results from a randomised, open-label, phase 3 trial. Lancet Oncol.

[13] Krop I.E., Kim S.-B., González-Martín A. (2014). Trastuzumab emtansine versus treatment of physician's choice for pretreated HER2-positive advanced breast cancer (TH3RESA): a randomised, open-label, phase 3 trial. Lancet Oncol.

[14] Krop I.E., Kim S.-B., Martin A.G. (2017). Trastuzumab emtansine versus treatment of physician's choice in patients with previously treated HER2-positive metastatic breast cancer (TH3RESA): final overall survival results from a randomised open-label phase 3 trial. Lancet Oncol.

[15] Lambertini M., Campbell C., Gelber R. (2019). Dissecting the effect of hormone receptor status in patients with HER2-positive early breast cancer: exploratory analysis from the ALTTO (BIG 2-06) randomized clinical trial. Breast Cancer Res Treat.

[16] Callahan R., Hurvitz S. (2011). HER2-positive breast cancer: current management of early, advanced, and recurrent disease. Curr Opin Obstet Gynecol.

[17] Di Cosimo S., Serpico D., Porcu L. (2014). Clinical outcome of HER2-positive breast cancer patients after failure on adjuvant trastuzumab: the potential of the time to relapse. Clin Oncol.

[18] Láng I., Bell R., Feng F.Y. (2014). Trastuzumab retreatment after relapse on adjuvant trastuzumab therapy for human epidermal growth factor receptor 2-positive breast cancer: final results of the Retreatment after HErceptin Adjuvant trial. Clin Oncol (R Coll Radiol).

[19] Lambertini M., Agbor-Tarh D., Metzger Filho O. (2020). Prognostic role of distant disease-free interval from completion of adjuvant trastuzumab in HER2-positive early breast cancer: analysis from the ALTTO (BIG 2-06) trial. ESMO Open.

[20] Giordano S.H., Temin S., Chandarlapaty S. (2018). Systemic therapy for patients with advanced human epidermal growth factor receptor 2–positive breast cancer: ASCO clinical practice guideline update. J Clin Oncol.

[21] Fabi A., De Laurentiis M., Caruso M. (2017). Efficacy and safety of T-DM1 in the “common-practice” of HER2+ advanced breast cancer setting: a multicenter study. Oncotarget.

[22] Griguolo G., Brasó-Maristany F., González-Farré B. (2020). ERBB2 mRNA expression and response to ado-trastuzumab emtansine (T-DM1) in HER2-positive breast cancer. Cancers (Basel).

[23] Conte B., Fabi A., Poggio F. (2020). T-DM1 efficacy in patients with HER2-positive metastatic breast cancer progressing after a taxane plus pertuzumab and trastuzumab: an Italian multicenter observational study. Clin Breast Cancer.

[24] Hammond M.E.H., Hayes D.F., Dowsett M. (2010). American Society of Clinical Oncology/College of American Pathologists guideline recommendations for immunohistochemical testing of estrogen and progesterone receptors in breast cancer. J Clin Oncol.

[25] Wolff A.C., Hammond M.E.H., Allison K.H. (2018). Human epidermal growth factor receptor 2 testing in breast cancer: American Society of Clinical Oncology/College of American Pathologists clinical practice guideline focused update. J Clin Oncol.

[26] Wolff A.C., Hammond M.E.H., Hicks D.G. (2013). Recommendations for human epidermal growth factor receptor 2 testing in breast cancer: American Society of Clinical Oncology/College of American Pathologists clinical practice guideline update. J Clin Oncol.

[27] Grambsch P.M., Therneau T.M. (1994). Proportional hazards tests and diagnostics based on weighted residuals. Biometrika.

[28] Schoenfeld D. (1982). Partial residuals for the proportional hazards regression model. Biometrika.

[29] Yamashiro H., Iwata H., Masuda N. (2020). Outcomes of trastuzumab therapy in HER2-positive early breast cancer patients: extended follow-up of JBCRG-cohort study 01. Breast Cancer.

[30] von Minckwitz G., Huang C.-S., Mano M.S. (2019). Trastuzumab emtansine for residual invasive HER2-positive breast cancer. N Engl J Med.

[31] Chumsri S., Li Z., Serie D.J. (2019). Incidence of late relapses in patients with HER2-positive breast cancer receiving adjuvant trastuzumab: combined analysis of NCCTG N9831 (Alliance) and NRG oncology/NSABP B-31. J Clin Oncol.

[32] Arpino G., Michelotti A., Truini M. (2016). Demographic, tumor and clinical features of clinical trials versus clinical practice patients with HER2-positive early breast cancer: results of a prospective study. J Cancer Res Clin Oncol.

[33] US Food and Drug Administration. Highlights of Prescribing Information for T-DM1. Available at: https://www.accessdata.fda.gov/drugsatfda_docs/label/2019/125427s105lbl.pdf. Accessed December 31, 2020.

[34] European Medicine Agency (2021). Summary of Product Characteristics of T-DM1. https://www.ema.europa.eu/en/documents/product-information/kadcyla-epar-product-information_en.pdf.

[35] Perez E.A., Barrios C., Eiermann W. (2017). Trastuzumab emtansine with or without pertuzumab versus trastuzumab plus taxane for human epidermal growth factor receptor 2–positive, advanced breast cancer: primary results from the phase III MARIANNE study. J Clin Oncol.

[36] Biskup E.M., Montavon Sartorius C., Müller A. (2019). Pertuzumab (P) as ≥ second-line therapy for HER2-positive metastatic breast cancer (mBC): Swiss clinical experience. Ann Oncol.

[37] Smyth L.M., Iyengar N.M., Chen M.F. (2016). Weekly paclitaxel with trastuzumab and pertuzumab in patients with HER2-overexpressing metastatic breast cancer: overall survival and updated progression-free survival results from a phase II study. Breast Cancer Res Treat.

[38] Bergin A.R.T., Luen S.J., Savas P. (2019). Efficacy of late line pertuzumab with trastuzumab and chemotherapy in HER2-positive metastatic breast cancer: an Australian case series. Asia Pac J Clin Oncol.

[39] Urruticoechea A., Rizwanullah M., Im S.-A. (2017). Randomized phase III trial of trastuzumab plus capecitabine with or without pertuzumab in patients with human epidermal growth factor receptor 2-positive metastatic breast cancer who experienced disease progression during or after trastuzumab-based therapy. J Clin Oncol.

[40] Del Prete S., Montella L., Arpino G. (2020). Second line trastuzumab emtansine following horizontal dual blockade in a real-life setting. Oncotarget.

[41] Vici P., Pizzuti L., Michelotti A. (2017). A retrospective multicentric observational study of trastuzumab emtansine in HER2 positive metastatic breast cancer: a real-world experience. Oncotarget.

[42] Battisti N.M.L., Rogerson F., Lee K. (2020). Safety and efficacy of T-DM1 in patients with advanced HER2-positive breast cancer The Royal Marsden experience. Cancer Treat Res Commun.

[43] Schettini F., Chic N., Brasó-Maristany F. (2021). Clinical, pathological, and PAM50 gene expression features of HER2-low breast cancer. NPJ Breast Cancer.

[44] von Minckwitz G., Procter M., de Azambuja E. (2017). Adjuvant pertuzumab and trastuzumab in early HER2-positive breast cancer. N Engl J Med.

[45] Gianni L., Pienkowski T., Im Y.-H. (2012). Efficacy and safety of neoadjuvant pertuzumab and trastuzumab in women with locally advanced, inflammatory, or early HER2-positive breast cancer (NeoSphere): a randomised multicentre, open-label, phase 2 trial. Lancet Oncol.

[46] US Food and Drug Administration. Highlights of Prescribing Information for Pertuzumab. Available at: https://www.accessdata.fda.gov/drugsatfda_docs/label/2013/125409s051lbl.pdf. Accessed December 31, 2020.

[47] European Medicine Aagency (2021). Summary of Product Characteristics of Pertuzumab. https://www.ema.europa.eu/en/medicines/human/EPAR/perjeta.

[48] Modi S., Saura C., Yamashita T. (2020). Trastuzumab deruxtecan in previously treated HER2-positive breast cancer. N Engl J Med.

[49] Rugo H.S., Im S.-A., Cardoso F. (2021). Efficacy of margetuximab vs trastuzumab in patients with pretreated ERBB2-positive advanced breast cancer: a phase 3 randomized clinical trial. JAMA Oncol.

[50] Murthy R.K., Loi S., Okines A. (2020). Tucatinib, trastuzumab, and capecitabine for HER2-positive metastatic breast cancer. N Engl J Med.

