# Supplementary material for: T-DM1 versus pertuzumab, trastuzumab and a taxane as first-line therapy of early-relapsed HER2-positive metastatic breast cancer: an Italian multicenter observational study
Source: ESMO Open. 2021 Apr 2;6(2):100099. doi: 10.1016/j.esmoop.2021.100099 (PMC8047485; doi:10.1016/j.esmoop.2021.100099)
Supplement: Supplementary Material [file mmc1.docx]

**Supplementary tables**

**Supplementary table 1. Study population’s clinicopathological features and treatment history according to TTR**

| **PATIENTS AND BASELINE TUMOR CHARACTERISTICS** | **TTR Groups** | | | | ***P*** |
| --- | --- | --- | --- | --- | --- |
|  | **≤6 months** | | **6-12 months** | |  |
|  | **N** | **%** | **N** | **%** |  |
|  | 53 | 70.7 | 22 | 29.3 |  |
| **Age at 1st-line** |  |  |  |  |  |
| *Median (years)* | 51 | - | 58 | - | **0.025** |
| *IQR (years)* | 42 - 57 | - | 50 - 63 | - |  |
| *Total* | 53 | 100.0 | 22 | 100.0 |  |
| **Sex** |  |  |  |  |  |
| *Female* | 53 | 100.0 | 22 | 100.0 | - |
| *Male* | 0 | 0.0 | 0 | 0.0 |  |
| *Total* | 53 | 100.0 | 22 | 100.0 |  |
| **Menopausal status** |  |  |  |  |  |
| *Pre/perimenopausal* | 20 | 38.5 | 5 | 23.8 | 0.232 |
| *Postmenopausal* | 32 | 61.5 | 16 | 76.2 |  |
| *Total* | 52 | 98.1 | 21 | 95.5 |  |
| **Histotype** |  |  |  |  |  |
| *Ductal* | 41 | 93.2 | 20 | 100.0 | 0.232 |
| *Lobular* | 3 | 6.8 | 0 | 0.0 |  |
| *Other* | 0 | 0.0 | 0 | 0.0 |  |
| *Total* | 44 | 83.0 | 20 | 90.9 |  |
| **T** |  |  |  |  |  |
| *1* | 8 | 19.5 | 10 | 47.6 | 0.111 |
| *2* | 18 | 43.9 | 7 | 33.3 |  |
| *3* | 7 | 17.1 | 1 | 4.8 |  |
| *4* | 8 | 19.5 | 3 | 14.3 |  |
| *Total* | 41 | 77.4 | 21 | 95.5 |  |
| **N** |  |  |  |  |  |
| *0* | 8 | 19.5 | 8 | 40.0 | 0.306 |
| *1* | 18 | 43.9 | 6 | 30.0 |  |
| *2* | 8 | 19.5 | 2 | 10.0 |  |
| *3* | 7 | 17.1 | 4 | 20.0 |  |
| *Total* | 41 | 77.4 | 20 | 90.9 |  |
| **HRs status** |  |  |  |  |  |
| *Positive* | 29 | 54.7 | 13 | 59.1 | 0.728 |
| *Negative* | 24 | 45.3 | 9 | 40.9 |  |
| *Total* | 53 | 100.0 | 22 | 100.0 |  |
| **Grading** |  |  |  |  |  |
| *G1-2* | 6 | 14.0 | 3 | 16.7 | 0.785 |
| *G3* | 37 | 86.0 | 15 | 83.3 |  |
| *Total* | 43 | 81.1 | 18 | 81.8 |  |
| **Ki67** |  |  |  |  |  |
| *<20%* | 5 | 10.9 | 5 | 23.8 | 0.168 |
| *≥20%* | 41 | 89.1 | 16 | 76.2 |  |
| *Total* | 46 | 86.8 | 21 | 95.5 |  |
| **Visceral metastases *ab initio*** |  |  |  |  |  |
| *Visceral (liver/lung)* | 25 | 50.0 | 9 | 40.9 | 0.477 |
| *Non-visceral (other from liver/lung)* | 25 | 50.0 | 13 | 59.1 |  |
| *Total* | 50 | 94.3 | 22 | 100.0 |  |
| **CNS metastases *ab initio*** |  |  |  |  |  |
| *CNS* | 11 | 21.2 | 7 | 31.8 | 0.328 |
| *Non-CNS* | 41 | 78.8 | 15 | 68.2 |  |
| *Total* | 52 | 98.1 | 22 | 100.0 |  |
| **(Neo)adjuvant HT** |  |  |  |  |  |
| *Yes* | 22 | 44.0 | 8 | 38.1 | 0.646 |
| *No* | 28 | 56.0 | 13 | 61.9 |  |
| *Total* | 50 | 94.3 | 21 | 95.5 |  |
| **HT type** |  |  |  |  |  |
| *Tamoxifen +/- GnRHa* | 8 | 40.0 | 5 | 62.5 | 0.281 |
| *AI +/- GnRHa* | 12 | 60.0 | 3 | 37.5 |  |
| *Other* | 0 | 0.0 | 0 | 0.0 |  |
| *Total* | 20 | 90.9 | 8 | 100.0 |  |
| **(Neo)adjuvant CT** |  |  |  |  |  |
| *Yes* | 48 | 90.6 | 19 | 86.4 | 0.591 |
| *No* | 5 | 9.4 | 3 | 13.6 |  |
| *Total* | 53 | 100.0 | 22 | 100.0 |  |
| **CT type** |  |  |  |  |  |
| *Anthracyclines without Taxanes* | 3 | 6.4 | 1 | 5.3 | 0.683 |
| *Taxanes without Anthracyclines* | 8 | 17.0 | 2 | 10.5 |  |
| *Anthracyclines + Taxanes* | 31 | 66.0 | 12 | 63.2 |  |
| *Other* | 5 | 10.6 | 4 | 21.1 |  |
| *Total* | 47 | 97.9 | 19 | 100.0 |  |
| **(Neo)adjuvant Trastuzumab** |  |  |  |  |  |
| *Yes* | 42 | 79.2 | 18 | 81.8 | 0.800 |
| *No* | 11 | 20.8 | 4 | 18.2 |  |
| *Total* | 53 | 100.0 | 22 | 100.0 |  |
| **First-line regimen** |  |  |  |  |  |
| *P+T+taxane* | 25 | 47.2 | 19 | 86.4 | **0.002** |
| *T-DM1* | 28 | 52.8 | 3 | 13.6 |  |
| *Total* | 53 | 100.0 | 22 | 100.0 |  |
| **Therapy after first-line PD** |  |  |  |  |  |
| *T-DM1* | 7 | 25.9 | 7 | 70.0 | **0.007** |
| *Lapatinib+capecitabine* | 15 | 55.6 | 0 | 0.0 |  |
| *Other* | 5 | 18.5 | 3 | 30.0 |  |
| *Total* | 27 | 50.9 | 10 | 45.5 |  |

**Legend.** P+T: pertuzumab + trastuzumab; HRs: hormone receptors; IQR: interquartile range; CNS: central nervous system. CT: chemotherapy; HT: hormone therapy; TTR: time-to-relapse; GnRHa: GnRH analogue; PD: progression of disease; *: percentages are based on the total of patients *per* arm.

**Supplementary table 2. Test for Schoenfeld residuals concerning multivariate Cox regression models for PFS and OS**

| **PFS Model Covariates** | **Chi square** | **Degrees of Freedom** | ***P*** |
| --- | --- | --- | --- |
| *Cohort* | 2.56 | 1 | 0.110 |
| *Age at 1st-line start* | 0.22 | 1 | 0.630 |
| *HRs status* | 0.05 | 1 | 0.822 |
| *(Neo)adjuvant CT* | 1.10 | 1 | 0.293 |
| *(Neo)adjuvant Trastuzumab* | 0.03 | 1 | 0.870 |
| *TTR* | 0.15 | 1 | 0.698 |
| *Visceral metastases* | 2.60 | 1 | 0.107 |
| *Brain metastases* | 2.58 | 1 | 0.108 |
| GLOBAL | 14.61 | 8 | 0.067 |
| **OS Model Covariates** | **Chi square** | **Degrees of Freedom** | ***P*** |
| *Cohort* | 0.16 | 1 | 0.690 |
| *Age at 1st-line start* | 0.86 | 1 | 0.350 |
| *HRs status* | 1.16 | 1 | 0.280 |
| *(Neo)adjuvant CT* | 2.45 | 1 | 0.120 |
| *(Neo)adjuvant Trastuzumab* | 1.29 | 1 | 0.260 |
| *TTR* | 0.22 | 1 | 0.640 |
| *Visceral metastases* | 0.52 | 1 | 0.470 |
| *Brain metastases* | 1.56 | 1 | 0.210 |
| GLOBAL | 10.91 | 8 | 0.210 |

**Legend.** CT: chemotherapy; TTR: time-to-relapse; HRs: hormone receptors; PFS: progression-free survival; OS: overall survival.

**Supplementary figures**

**Supplementary figure 1. STROBE flow-chart**

**Legend.** *: only HER2+ patients were considered; P+T: pertuzumab + trastuzumab; TTR: time-to-relapse.

**Supplementary figure 2. Post-progression survival according to 1^st^-line treatment**

**Legend.** Kaplan-Meier curves with respective 95% confidence limits; P+T: pertuzumab + trastuzumab.

**Supplementary figure 3. Progression-free survival and overall survival in the pertuzumab-treated cohort according to TTR**

**Legend.** Progression-free survival (A) and overall survival (B) Kaplan-Meier curves with respective 95% confidence limits of P+T+taxane in the population with TTR 6-12 months vs TTR≤6 months. P+T: pertuzumab+trastuzumab; TTR: time-to-relapse.

**Supplementary figure 4. PFS Schoenfeld residuals plots**

**Legend.** A: covariate treatment cohort; B: covariate upfront visceral metastases; C: covariate upfront brain metastases; D: covariate HRs status; E: covariate (neo)adjuvant chemotherapy; F: covariate (neo)adjuvant trastuzumab; G: covariate TTR; H: covariate age at 1^st^-line ; HRs: hormone receptors; TTR: time-to-relapse; PFS: progression-free survival.

**Supplementary figure 5. OS and PPS Schoenfeld residuals plots**

**Legend.** A: covariate treatment cohort; B: covariate upfront visceral metastases; C: covariate upfront brain metastases; D: covariate HRs status; E: covariate (neo)adjuvant chemotherapy; F: covariate (neo)adjuvant trastuzumab; G: covariate TTR; H: covariate age at 1^st^-line; I: covariate treatment cohort, for PPS; HRs: hormone receptors; TTR: time-to-relapse; OS: overall survival; PPS: post-progression survival.
